# Editorial: Leveraging phenotyping and crop modeling in smart agriculture

**DOI:** 10.3389/fpls.2025.1626622

**Published:** 2025-07-03

**Authors:** Ting Sun, Liujun Xiao, Syed Tahir Ata-Ul-Karim, Yuntao Ma, Wenyu Zhang

**Affiliations:** ^1^ Jiangsu Academy of Agricultural Sciences Wuxi Branch, Wuxi, Jiangsu, China; ^2^ National Engineering and Technology Center for Information Agriculture, Key Laboratory for Crop System Analysis and Decision Making, Jiangsu Collaborative Innovation Center for Modern Crop Production, Nanjing Agricultural University, Nanjing, Jiangsu, China; ^3^ Department of Agroecology, Climate and Water, Aarhus University, Tjele, Denmark; ^4^ College of Land Science and Technology, China Agricultural University, Beijing, China

**Keywords:** phenotyping, modeling, smart agriculture, functional-structural plant models, environment, genotype

In recent years, the agricultural sector has witnessed a significant transformation driven by the integration of sensing technologies, big data analytics, and artificial intelligence (Ahmed and Shakoor, 2025). Cutting-edge innovations, notably high-throughput phenotyping and crop modeling, have fundamentally altered our understanding and management of crop systems (Keating and Thorburn, 2018; Yang et al., 2020). In many cases, phenotyping and modeling are closely intertwined: phenotyping provides accurate characterization of plant traits, forming the basis for reliable crop models, while modeling elucidates interactions among phenotypes, genotypes, and the environment, and enables prediction of phenotypic outcomes (Yu et al., 2023; Zhang et al., 2023b). Despite their natural synergy, phenotyping and modeling are still frequently treated as separate domains, limiting their full potential. This Research Topic aims to close that gap by promoting the development of integrated phenotyping-modeling frameworks to advance smart agriculture. The following sections provide a categorized overview of the contributions to this Research Topic (https://www.frontiersin.org/research-topics/62521/leveraging-phenotyping-and-crop-modeling-in-smart-agriculture), highlighting key findings and identifying future directions for this rapidly advancing field.

## 1 Crop phenotyping

Crop phenotyping, which plays a vital role in gene function analysis, plant breeding, and smart agriculture, can be broadly categorized based on the traits measured. Morphological and structural traits include leaf length, leaf width, leaf area, and leaf angle, while physiological and biological traits encompass chlorophyll content, nitrogen levels, transpiration, and photosynthetic parameters.

### 1.1 Morphological and structural phenotypes

2D imaging combined with machine vision remains the most widely adopted technique for acquiring plant morphological and structural phenotypes. In this topic, a range of studies have explored deep learning-based approaches tailored for specific plant phenotyping applications, with a particular focus on refining model architectures and technical strategies to enhance detection accuracy, computational efficiency, and adaptability to complex field conditions. Among them, semantic segmentation frameworks have been effectively employed for fine-grained plant disease identification. Ding et al. employed DeepLabV3+ with an integrated Attention Pyramid Fusion (APF) module, achieving rapid and accurate segmentation of sweetpotato virus disease (SPVD) lesions in field images. Building on the success of YOLO-based object detection, several teams developed tailored variants to tackle specific phenotyping challenges. Pan et al. developed KIWI-YOLO, a kiwifruit flower pollination detection model based on YOLOv5, incorporating a frequency domain feature fusion (FDFF) module and a Bi-Level Routing Attention (BRA) mechanism to improve feature focus and detection performance. Qiu et al. introduced YOLO-SDL, which combines the lightweight YOLOv8n framework with a ShuffleNetV2 backbone and integrates depthwise separable convolutions (DWConv) and the large separable kernel attention (LSKA) module in the neck to enhance wheat grain classification while ensuring compactness and computational efficiency. Wu et al. proposed TiGra-YOLOv8, which integrates an Attentional Scale Fusion (ASF) module, Adaptive Training Sample Selection (ATSS), and Wise-IoU loss, while employing channel pruning to optimize model size and inference speed for grapefruit detection in dense orchard environments. Wang et al. presented PDSI-RTDETR for tomato ripeness detection, improving RT-DETR by replacing the Basic_Block with PConv_Block and integrating deformable attention with intra-scale feature interaction. The model also introduces a slimneck-SSFF fusion structure and replaces GIoU with Inner-EIoU loss to accelerate convergence and improve small object detection accuracy. Zhang et al. proposed YOLOv8-FCS for grading fingered citron slices, enhancing YOLOv8n by substituting its backbone with the Fasternet module and redesigning the PAN-FPN structure using BiFPN to improve computational efficiency and multi-scale feature utilization. Qing et al. also contributed an improved YOLO-FastestV2 model for wheat spike detection, incorporating a multi-stage attention mechanism and LightFPN detection head to optimize detection under variable field conditions. Beyond object detection, several studies addressed plant organ counting through CNN-Transformer hybrid models and lightweight convolutional networks. Hong et al. introduced CTHNet, which combines a CSP-based CNN for multi-scale local feature extraction with a Pyramid Pooling Transformer for global context learning, further enhanced by a feature fusion module to improve wheat ear counting performance. Yang et al. developed a wheat ear positioning and counting approach based on FIDMT-GhostNet, leveraging GhostNet for multi-scale feature extraction, a dense upsampling module for improved image resolution, and a local maximum detection strategy to reduce background noise and enhance counting accuracy in dense environments. Complementing these RGB-based imaging approaches, UAV-based hyperspectral imaging has also been utilized for monitoring canopy structural traits. Fan et al. proposed a potato leaf area index (LAI) estimation model by integrating spectral and textural features selected via the Successive Projection Algorithm (SPA) with machine learning, finding that spectral data exhibited higher sensitivity to LAI than Haralick texture features. Collectively, these studies highlight the diverse technical strategies that are accelerating plant phenotyping. Through architectural refinements and targeted module innovations, they offer efficient, accurate, and scalable solutions suited to real-world, high-throughput agricultural applications.

2D image-based methods are inherently constrained by issues such as occlusion, perspective distortion, and the loss of spatial information. In contrast, 3D approaches are capable of capturing the complete spatial geometry and topological structure of plants, offering a richer and more precise foundation for the analysis of complex phenotypic traits. Nevertheless, reconstructing and analyzing plant 3D point clouds remains challenging due to dense occlusions, intricate structural details, complex organ overlaps, and variations in lighting and background conditions. Overcoming these challenges is critical for advancing high-throughput, *in-situ* plant phenotyping and digital twin modeling in agricultural research. Song et al. gathered 1,431 ear leaves from 518 maize inbred lines at the silking stage using a 3D digitizer. Area-preserving 2D leaf models were generated through mesh subdivision and planar parameterization. Eleven semantic features were identified via clustering and correlation analysis. A 2D leaf shape indicator (L2D) and an atlas were developed, allowing precise identification of inbred lines based on 2D leaf shape. Rodriguez-Sanchez et al. developed a spatiotemporal registration approach for time-series terrestrial laser scanning data, enabling continuous 4D monitoring of cotton canopy traits with high spatial accuracy, and used the registered models to track growth dynamics and assess genotype differences throughout the season. Although 3D digitizers, LiDAR, and similar technologies have been widely applied for crop 3D reconstruction, their high costs and limited precision have significantly constrained broader agricultural applications. In recent years, with the decreasing cost of industrial-grade cameras, multi-view stereo (MVS) based 3D reconstruction has emerged as a mainstream solution. Wu et al. developed a fast and accurate 3D reconstruction platform for the mandarin orange based on Object-Based NeRF (OB-NeRF). By integrating optimized camera pose calibration, efficient ray sampling, and exposure adjustment, the platform reconstructs high-quality neural radiance fields from videos within 250 seconds. Sun et al. employed a multi-view imaging platform to capture wheat plant images, generating high-quality point clouds through Structure-from-Motion and Multi-View Stereo (SfM-MVS) using Euclidean clustering, color filtering, and statistical methods. A region-growing algorithm was used for stem and leaf segmentation, though substantial leaf overlap during the tillering, jointing, and booting stages made the process particularly challenging. Plant height, convex hull volume, plant surface area, and crown area were extracted, enabling a detailed analysis of dynamic changes in wheat throughout its growth cycle. In recent years, ultra-low-altitude UAV-based cross-circling oblique imaging has become a more efficient and cost-effective approach for in-field 3D reconstruction (Fei et al., 2025; Sun et al., 2024). Unlike indoor multi-view imaging systems, 3D phenotyping conducted directly in the field more accurately reflects real-world agricultural conditions and population-level dynamics.

### 1.2 Physiological and biological phenotypes

Physiological and biological phenotypes are typically assessed rapidly and non-destructively using hyperspectral or multispectral techniques. Yang et al. combined fractional-order derivatives (FOD) with machine learning techniques to estimate chlorophyll density in winter wheat using hyperspectral imagery. Three FOD methods and eight machine learning models were tested with both full-spectrum data and CARS-selected bands. The Riemann-Liouville FOD (RL-FOD) showed superior performance in model construction. The highest accuracy was achieved by combining 0.3-order RL-FOD, CARS-based band selection, and extra-trees regression (ETsR). Su et al. used UAV multispectral sensors to capture winter wheat canopy images, extracting spectral and texture features. Feature selection methods (Boruta and Recursive Feature Elimination) were applied to identify key features, and a feature fusion strategy combined with Support Vector Machine Regression was used to develop the SPAD estimation model. The results indicated that combining NIR spectral features with other bands, along with red and NIR texture features, effectively captured SPAD variations during the reproductive growth stage. Jiang et al. presented a ChlF dataset of hydroponic lettuce seedlings, consisting of transient images captured under different cultural conditions. The effectiveness of the threshold segmentation algorithm and the Deeplabv3+ algorithm for extracting the seedling canopy was compared. Sun et al. utilized UAV hyperspectral and ultra-high-resolution RGB images to derive vegetation indices, texture features, and structural characteristics for estimating rapeseed aboveground biomass. Various models, including deep neural networks, random forests, and support vector regression, were tested with different feature combinations. Models that incorporated all three feature types delivered higher accuracy compared to those using individual feature sets, with deep neural networks consistently outperforming the other algorithms. Luo et al. reviewed the advancements in applying hyperspectral imaging technology to obtain information on tea plant phenotypes, growth conditions, and quality indicators under environmental stress. Wang et al. applied multi-leaf SPAD measurements combined with machine learning to improve nitrogen diagnostics in rice. Integrating SPAD data with models like Random Forest and Extreme Gradient Boosting enhanced the estimation accuracy of Leaf Nitrogen Concentration (LNC) and Nitrogen Nutrition Index (NNI). The second leaf from the top was most important for predicting LNC, while the third leaf was key for NNI. Shi et al. evaluated flavonoid content (Flav) and the Nitrogen Balance Index (NBI), measured by a Dualex sensor, alongside machine learning models for nitrogen status assessment. Data from 15 rice varieties under varying nitrogen rates showed chlorophyll saturation at high nitrogen levels, while Flav and NBI remained reliable. Random Forest and Extreme Gradient Boosting achieved high prediction accuracy, with SHAP analysis identifying NBI and Flav from the top two leaves as critical predictors. In recent years, these technologies have been widely applied to precision farmland management. For example, on farms in Brazil, Castilho Silva et al. (2025) used UAV-based multispectral remote sensing to monitor leaf nitrogen content in maize and applied variable-rate fertilization accordingly. Compared to conventional methods, this approach reduced nitrogen input by 6.6% to 35% without compromising yield.

### 1.3 Phenotyping equipment

Phenotyping equipment is essential for the precise monitoring of plant traits and environmental growth conditions. Liu et al. developed a portable vegetation canopy reflectance (VCR) sensor for continuous operation throughout the day, featuring optical bands at 710 nm and 870 nm. The sensor was calibrated using an integrating sphere and a solar altitude correction model, with validation against a standard reflectance gray scale board. Field measurements taken at 14 sites using both the VCR sensor and an ASD spectroradiometer showed closely aligned reflectance values. In Bermuda grass measurements, the intra-day reflectance range narrowed and the coefficient of variation decreased after solar altitude correction, demonstrating the sensor’s effectiveness for precise vegetation monitoring. Compared to remote sensing, recent developments in flexible sensors enable direct, continuous, and high-resolution monitoring of plant physiological traits and environmental conditions (Zhang et al., 2024). These innovative sensing technologies are poised to significantly enhance phenotyping applications.

## 2 Crop modeling

While various models for the direct extraction or inversion of crop phenotypes have been explored in the crop phenotyping section, crop modeling in this context specifically refers to growth modeling designed to predict crop development and growth. Depending on the approach, crop growth models may be data-based, incorporating machine learning techniques, or mechanistic, based on process-based simulations of crop physiological processes (Maestrini et al., 2022). In this topic, process-based models are limited, with more researchers focusing on simpler modeling approaches.

### 2.1 Data-based models

Takahashi et al. proposed a machine learning approach for early prediction of tomato fruit size at harvest, comparing Ridge Regression, Extra Tree Regression, and CatBoost Regression models. Estimated fruit weight, derived from diameter measurements at various cumulative temperatures after anthesis, was used as the explanatory variable, with final harvest weight as the target. Results indicated that incorporating estimates from multiple cumulative temperature points improved prediction accuracy, particularly for cultivars with stable growth patterns. Including average temperature as a variable further enhanced model performance. Yang et al. compared classical non-linear models and deep learning methods for predicting alfalfa leaf area index (LAI). Logistic, Gompertz, and Richards models were developed based on growth days, while a time-series model integrating environmental factors was proposed using a mutation point detection method and an encoder-attention-decoder BiLSTM network (TMEAD-BiLSTM). Results showed that the TMEAD-BiLSTM model outperformed non-linear models in prediction accuracy and effectively integrated environmental factors. Nian et al. estimated the rice aboveground biomass based on the first derivative spectrum and Boruta algorithm. Mustafa et al. developed a knowledge framework for yield prediction in cereal crops by leveraging UAVs.

### 2.2 Data assimilation between phenotyping and crop models

Gao et al. explored the quantitative relationship between soil profile salinity and soil depth in drip-irrigated cotton fields in southern Xinjiang using a multivariate linear regression model combined with a Kalman filter algorithm. The model effectively captured the dynamic changes in soil salinity across different growth stages and improved prediction accuracy after data assimilation. Based on the calibrated model and predicted soil conductivity data, the total cotton yield and income in the study area were estimated. The results demonstrate that the Kalman filter can enhance model reliability, providing a practical tool for monitoring soil salinity dynamics, assessing the relationship between soil salinity and cotton yield, and supporting efficient saline soil management in cotton fields. Zhou et al. propose a method for fruit selection and location in harvesting robots that accounts for obstacle perception. Synthetic data were generated using a 3D tomato greenhouse model and pixel-level segmentation labels. An attention-based feature extraction module (SFM) was designed to enhance the DeepLab v3+ segmentation network, improving detection of linear obstructions like stems and wires. An adaptive K-means clustering method was used to identify individual fruits. The barrier-free fruit selection algorithm identifies the largest, non-occluded fruit as the optimal target. This approach effectively detects and locates barrier-free fruits, providing a reliable solution for harvesting robots, applicable to other fruits and vegetables as well.

## 3 Perspectives

**Figure 1:**
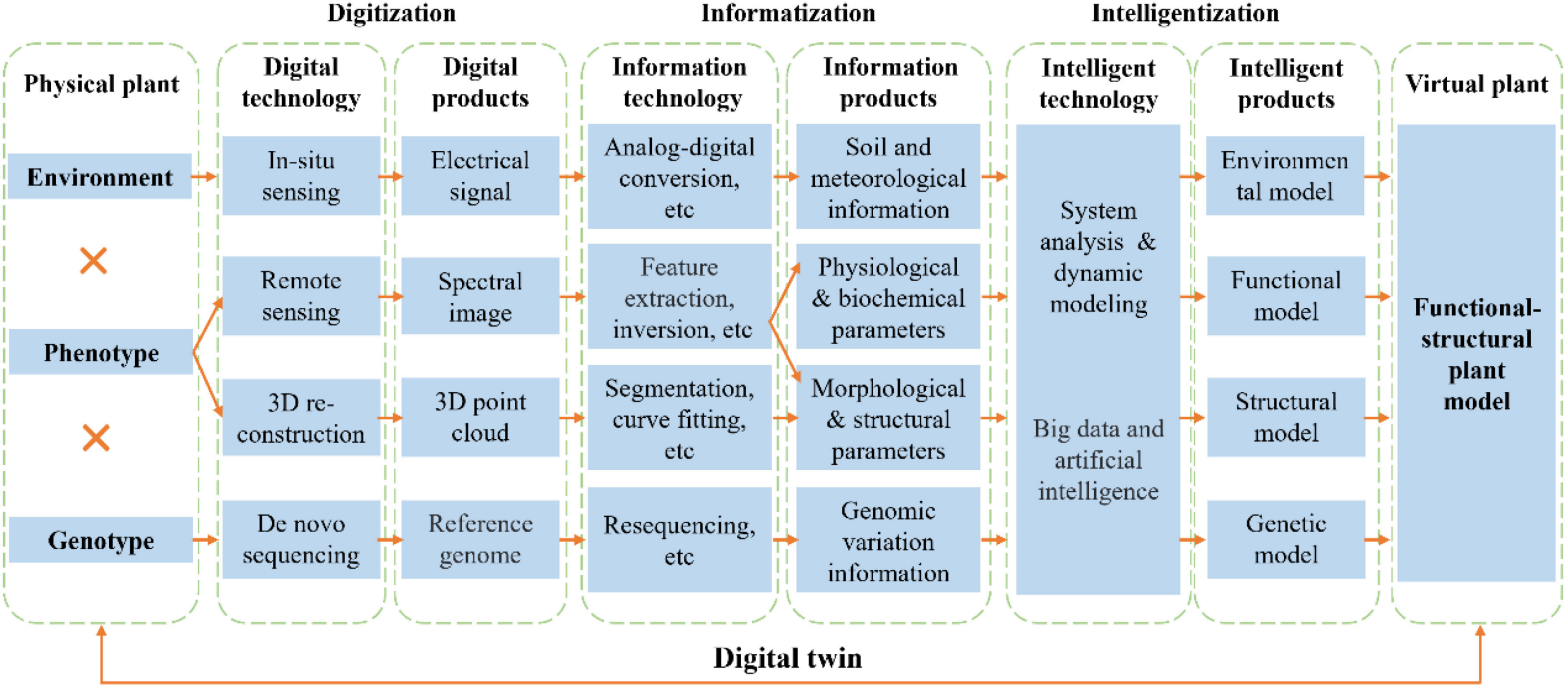
An integrated framework linking plant phenotype, genotype, and environment. The diagram outlines the collection of physiological, biochemical, morphological, structural, genomic, soil, and meteorological data using rapidly evolving technologies in recent years. These data are integrated through functional–structural plant models to link the physical and virtual representations of the plant, enabling the creation of a digital twin.

In conclusion, we propose an integrated framework that links plant phenotype, genotype, and environment (**Figure 1**), aiming to better synthesize current research efforts. Environmental parameters are commonly obtained via *in-situ* sensing, where sensors capture electrical signals (e.g., capacitance, resistance) and convert them into quantitative data such as air temperature, humidity, atmospheric pressure, photosynthetically active radiation, and soil temperature and moisture. These parameters facilitate the development of microclimate models, which can be further coupled with other simulation models. Phenotypic information is generally acquired through remote sensing and 3D reconstruction. Multispectral or hyperspectral imagery is processed through feature extraction and inversion to retrieve physiological and biochemical traits, while RGB imagery enables extraction of morphological and structural features at 2D level. Additionally, 3D point clouds derived from LiDAR or multi-view image reconstruction are processed through segmentation and surface modeling to obtain 3D structural traits. Therefore, functional and structural models are established through system analysis and dynamic modeling based on these phenotypes. In recent years, such models have been widely applied to investigate the impacts of climate change on crop productivity, identify potential yield gaps, and explore targeted improvement pathways (Gavasso-Rita et al., 2023). On the genetic level, reference genomes from *de novo* sequencing and genomic variations from resequencing support the development of genotype-based models (Zhang et al., 2023a).The integration of big data and artificial intelligence further enables hybrid modeling approaches—such as knowledge-guided machine learning (KGML) (Li et al., 2025) and improved phenotype–model data assimilation techniques (Jin et al., 2018). KGML leverages mechanistic knowledge of biological processes to guide the learning process, enhancing model interpretability and generalization capacity. Meanwhile, data assimilation techniques dynamically update model states and parameters using real-time phenotypic observations, thereby allowing high-throughput phenotyping data acquired by modern sensing technologies to be effectively integrated into the modeling framework. This unified phenotyping-modeling framework creates a digital twin by linking physical plants to their virtual counterparts, offering a promising pathway to integrate phenotyping with modeling for intelligent breeding and smart agriculture. However, the current framework remains incomplete, as it primarily emphasizes the virtual simulation of physical plants. Achieving a true digital twin requires establishing reverse control mechanisms that enable real-time feedback from the virtual twin to the physical system—a process that depends on further advancements in intelligent agricultural equipment and the seamless integration of agronomic practices with agricultural machinery.
